# A process evaluation of a "physical activity pathway" in the primary care setting

**DOI:** 10.1186/1471-2458-10-463

**Published:** 2010-08-09

**Authors:** Fiona C Bull, Karen E Milton

**Affiliations:** 1 School of Population Health, The University of Western Australia, 35 Stirling Highway, Crawley WA 6009, Australia; 2 British Heart Foundation National Centre for Physical Activity and Health, School of Sport, Exercise and Health Sciences, Loughborough University, Epinal Way, Leicestershire, LE11 3TU, UK

## Abstract

**Background:**

Let's Get Moving (LGM) is a systematic approach to integrating physical activity promotion into the primary care setting. LGM combines a number of recommended strategies to support behavior change including brief interventions, goal-setting, written resources, and follow-up support. This study involved a process evaluation of implementing LGM in UK general practice.

**Methods:**

The LGM intervention was implemented in six general practices in London. Practices recruited patients either 'opportunistically' in routine consultations or by letter of invitation sent to patients on the hypertension disease register. A key component of the intervention was the delivery of a brief counselling session aimed at facilitating physical activity behaviour change. Data collection methods included electronic patient records, a practice survey and focus groups and interviews with practitioners.

**Results:**

A total of 526 patients were considered for LGM, 378 via the 'opportunistic' recruitment method and 148 using the disease register approach. Patient interest in the brief counselling session was high although the actual delivery style and content varied between practitioners. Patients were directed towards a variety of physical activity opportunities including local leisure services and walking schemes.

**Conclusion:**

The learning from this pilot should inform a revised update of the LGM protocols before the planned dissemination of the intervention which is outlined in the Governments 'Be Active, Be Healthy' physical activity strategy. A robust assessment of effectiveness involving an experimental design and behaviour change measures is also warranted prior to wider dissemination.

## Background

Participation in regular physical activity is associated with the prevention of chronic disease and the promotion of health and well-being[1]. Despite these benefits, national data show that in England 60% of men and approximately 70% of women are insufficiently active to benefit their health[2]. The estimated annual cost of physical inactivity in England is between £1 billion and £1.8 billion, with an average annual cost of £5 million for each Primary Care Trust[3].

Promoting physical activity in primary care is recognised as an important and potentially effective approach[4-6]. In recent years a number of initiatives have been tested in the primary care setting including advice and counselling [7-9], the provision of written resources [10] and referral to structured exercise programmes[6,11].

In the UK, exercise referral (ER) is the most wide-spread approach to promoting physical activity in primary care, with over 600 schemes [6] providing 91% of primary care organisations with access to an ER scheme[12]. Exercise referral was initially developed as part of the treatment plan for patients with existing health problems. With the rapid expansion of ER schemes, the inclusion criteria has broadened, such that many schemes now include low risk patients who may not require the level of supervision provided by exercise referral. In order to provide an effective and cost efficient approach to physical activity promotion, a system that includes protocols for low risk patients is needed.

**Figure 1:**
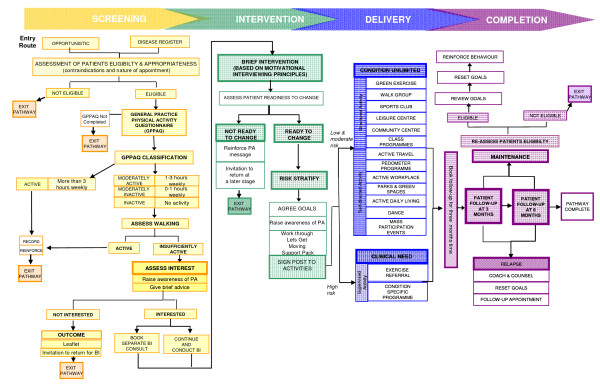
**Schematic diagram of the Let's Get Moving intervention**.

In 2007, the Department of Health in England developed Let's Get Moving (LGM), a systematic approach to integrating physical activity into primary care, for all patients ranging from low to high risk (Figure 1). LGM combines a number of recommended strategies to support behavior change, including brief interventions, goal-setting, written resources, and follow-up support[6,13]. A central component of LGM is the brief intervention aimed at providing advice to facilitate behaviour change. The inclusion of the brief intervention (BI) was based on review level evidence and subsequent guidance issued by the National Institute for Health and Clinical Excellence, which identified brief interventions within primary care as effective[6].

Although there is support for the use of physical activity counselling in health care settings [14] there is a lack of consensus in terms of what this counselling should consist of and how it should be delivered. Motivational interviewing (MI) has been identified as one potential approach to assist patients in changing lifestyle related risk factors and although research on effectiveness is equivocal [9,15-17] MI was selected for use in LGM.

This paper reports results from a process evaluation of LGM in a sample of UK general practices. The evaluation objectives were: to assess two patient recruitment methods ('opportunistic' and via disease registers); to track patients through the intervention and assess delivery of each component; to assess the time requirements of delivery; and to collect qualitative feedback from practitioners on their experiences of implementation.

## Methods

LGM was tested in a convenient sample of six general practices across London between March and August 2008. Practices were selected from a range of localities, to reflect diversity in socio-economic status and patient demographics such as ethnicity. At one practice, almost 90% of registered patients were Asian or Asian British.

A total of 10 practitioners (GP's, nurses, health care assistants) attended a two-day LGM training course. After which, practices were requested to recruit patients over a 12 week period, either 'opportunistically' (n = 3) or by targeting patients on their hypertension disease registers (n = 3). Practices recruiting 'opportunistically' were asked to consider every patient for LGM during routine consultations; disease register sites recruited patients via a letter of invitation sent to patients' home addresses. Patient inclusion criteria were: aged 16-74 years; no contra-indications to exercise; not meeting recommended levels of physical activity; and, for 'opportunistic' practices, it was appropriate to discuss physical activity within the context of the consultation.

Physical activity levels were assessed using the GPPAQ (General Practice Physical Activity Questionnaire) [18] which classifies patients on a Physical Activity Index (PAI; Table 1). The PAI does not include the amount of walking undertaken, however in this study practitioners were encouraged to discuss reported walking with patients and if appropriate, modify the patients' PAI. Patients who were not classified as 'active' were eligible to receive the brief intervention (BI), and this could be provided either as an extension to the screening (recruitment) consultation or booked as a separate appointment.

**Table 1:** The four-level Physical Activity Index for the GPPAQ and eligibility for LGM

Eligibility for LGM	PAI	Activity Level	Number of participants in each PAI category*
✗	**Active** (More than 3 hours weekly)	Sedentary job and ≥ 3 hours physical exercise and/or cycling per week OR	126 (28%)
		Standing job and 1-2.9 hours physical exercise and/or cycling per week OR	
		Physical job and some but < 1 hour physical exercise and/or cycling per week OR	
		Heavy manual job	

✓	**Moderately active** (1 - 3 hours weekly)	Sedentary job and 1-2.9 hours physical exercise and/or cycling per week OR	31 (7%)
		Standing job and some but < 1 hour physical exercise and/or cycling per week OR	
		Physical job and no physical exercise or cycling	

✓	**Moderately inactive** (0 - 1 hours weekly)	Sedentary job and some but < 1 hour physical exercise and/or cycling per week OR	90 (20%)
		Standing job and no physical exercise or cycling	

✓	**Inactive** (No activity reported)	Sedentary job and no physical exercise or cycling	198 (45%)

*PAI data were missing for 4 patients

The purpose of the BI was for the practitioner to utilise adapted motivational interviewing methods to enhance patients' willingness and confidence to change their physical activity behaviour. The BI involved discussing the importance and benefits of physical activity, goal setting and directing (or 'signposting') patients to local physical activity opportunities.

Practitioners used set criteria to assess the potential risk to each patient of taking part in physical activity based on their disease status. Protocols for patients identified as 'high risk' indicated supervised activity such as ER schemes. 'Medium' and 'low risk' patients could be directed towards a variety of opportunities including structured (e.g., health walks, sports clubs, and local leisure facilities) and self-directed activities (e.g., pedometer loan schemes and 'green exercise'). Although 'high risk' patients were restricted to clinically supervised activity, the underlying principle of the 'signposting' was that decisions were made in collaboration with the patient.

All patients were given a resource booklet containing information on the benefits of physical activity, details of local physical activity opportunities, and a local area map. The LGM protocols specified patient follow-up consultations at three and six months, however due to the timelines of the pilot study, practices were asked to undertake a three month follow-up only. The purpose of the follow-up consultation was to provide on-going support to facilitate sustained behaviour change.

The evaluation framework involved data collection from multiple sources. Patient recruitment and progress through the LGM intervention was tracked by data recorded in the Egton Medical Information System (EMIS) or similar practice software systems. EMIS captured data on which components of the intervention were delivered to each patient and the estimated time taken. These data were entered by practitioners during or directly after each patient consultation.

Data were downloaded using a MIQUEST search which extracted all data recorded on the EMIS templates as well as selected patient demographics including age, gender and ethnicity. In addition, each practice was asked to record details of any additional tasks undertaken to deliver LGM (e.g. writing letters to patients and arranging follow-up appointments). A short survey was provided to collect these data.

One focus group discussion was undertaken with five practitioners to capture their views and experiences of implementation. A semi-structured interview schedule was developed with questions on the GPPAQ, use of MI techniques, and recommendations for improvements to the LGM protocols. Additional telephone interviews (n = 5) were conducted after completion of the pilot study using a semi-structured questionnaire to further explore apparent differences between practices in the delivery of LGM. Focus group and telephone discussions were recorded on a digital audio device.

EMIS data were available from all six practices for the screening phase of the intervention, but from only five practices for the BI and follow-up stages. One practice omitted to use the correct EMIS template for the BI (practice #6) and one other practice did not complete EMIS for the follow-up consultations (practice #4). Descriptive statistics were used to report patient recruitment rates and the frequency of delivery of each component of the intervention. The focus group and interview data were transcribed and deductive content analysis was undertaken utilising the key components of LGM as the guiding themes. This type of deductive reasoning has been identified as appropriate for use when generating concepts or variables based on existing theory or knowledge[19,20].

The National Research Ethics Service (NRES) advised that this pilot, including its evaluation, falls within the category of 'audit' and did not require local ethics committee (LEC) approval.

## Results

Across the three 'opportunistic' practices, 378 patients were assessed for eligibility. Using the conservative estimate that over the 12 week recruitment period, approximately 5,900 consultations were conducted, this gives an estimated recruitment rate of 6%. Qualitative feedback from practitioners indicated that they made their own subjective appraisal of a patient's suitability for LGM based on perceived level of motivation and interest, and thus selected patients who they considered most likely to adhere to the intervention. Three practices recruited via hypertension disease registers and 916 letters were sent to patients. A total of 148 patients accepted the invitation, and the response rate varied markedly (9%, 12% and 59%).

Across all six practices a total of 526 patients were considered for the LGM intervention. Table 2 shows the number of patients screened at each practice and Table 3 shows the demographics of patients. More females (57%) were screened than males (43%) and the mean age of patients was 54 years. Just over half of patients (52%) were Asian or Asian British and 19% were White. Ethnicity data for 24% of patients were missing from practice records.

**Table 2:** Number of patients progressing to each stage of the intervention by practice

Practice	1	2	3	4	5	6	ALL
Recruitment Method	DR*	DR*	OPP^∓^	DR*	OPP^∓^	OPP^∓^	

Number of health professionals	2	1	2	2	2	1	10

Number of patients screened	28	49	114	71	220	44	526

Number of patients receiving BI	25	43	62	65	119	---	314

Number of patients attending follow-up	10	11	16	---	38	26	101

* Disease register

^∓ ^opportunistic

**Table 3:** Demographics of patients assessed for Let's Get Moving

Practice	1	2	3	4	5	6	ALL
**Age**							
Range	43-74	34-73	10-84	41-74	16-87	15-88	10-88
Mean	59.50	61.20	50.63	56.52	52.86	44.23	53.68
SD	8.75	8.76	15.24	8.78	16.13	15.80	14.88

**Gender**							
Male	11	18	47	29	105	14	224
Female	17	31	67	42	115	30	302

**Ethnicity**							
White	13 (46%)	35 (71%)	7 (6%)	12 (17%)	19 (9%)	13 (30%)	99 (19%)
Black or Black British	1 (4%)	2 (4%)	1 (1%)	8 (11%)	9 (4%)	7 (16%)	20 (4%)
Asian or Asian British	14 (50%)	9 (18%)	102 (89%)	1 (1%)	126 (57%)	15 (34%)	274 (52%)
Mixed	0	1 (2%)	1 (1%)	0	0	1 (2%)	3 (1%)
Other	0	2 (4%)	0	1 (1%)	0	0	3 (1%)
Unknown	0	0	0	0	0	1 (2%)	1 (0%)
Missing	0	0	3 (3%)	50 (70%)	66 (30%)	7 (16%)	126 (24%)

**Figure 2:**
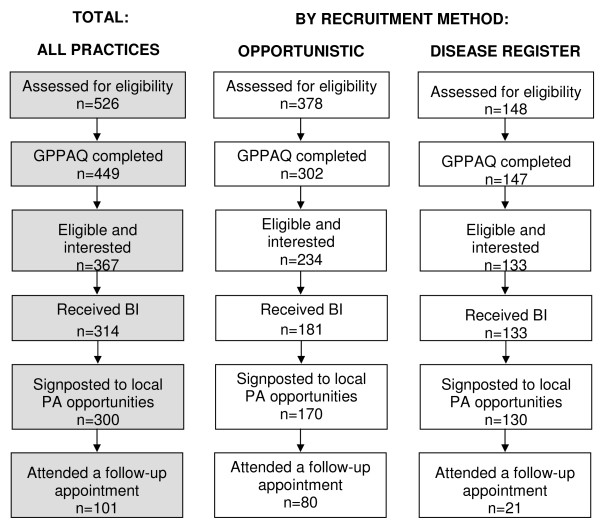
**Tracking of patients through the Let's Get Moving intervention for the whole sample and by recruitment method**.

Figure 2 shows the patient flow through the LGM intervention and the number of patients receiving each component. Of the 526 patients considered for LGM, 20 were deemed inappropriate due to their age, displaying contra-indications to exercise, or it was not considered appropriate to discuss physical activity within the context of the consultation. A further 57 patients were not asked to complete the GPPAQ due to time constraints and took no further part in the intervention. A total of 449 patients completed the GPPAQ, of which 319 (71%) were categorised as less than 'active' and thus eligible for LGM (see Table 1). Another 48 patients, classified as 'active' on GPPAQ, expressed an interest in LGM and were also included in the study despite being ineligible. Health practitioners reported that the GPPAQ was a practical tool for screening patients as it comprised relatively few questions, was easy to understand and took less than two minutes to complete.

A total of 314 patients received the brief intervention. However, this figure likely under-estimates the proportion of patients progressing to this stage of the intervention due to missing data from one practice (practice #6). Only 75 patients were not interested in the BI, the majority of whom were from Asian or Asian British ethnic groups. This suggests there are additional barriers to participation in LGM among these population groups and that more targeted recruitment may be necessary to engage these patients.

The majority of BI's were conducted within the initial screening consultation, rather than booked as a separate appointment. This was almost always the case in disease register practices (96%), most likely because practitioners and patients were aware of the purpose of the consultation and consequently booked a double-appointment. Surprisingly, the majority of patients recruited 'opportunistically' also received the screening and BI during the same consultation (75%), suggesting less interest or need for a second appointment.

EMIS data showed that overall each component of the BI was provided to the majority of patients, including a discussion on the benefits of physical activity (n = 313), goal setting (n = 295), and signposting to local physical activity opportunities (n = 300). However, practitioner feedback indicated that the delivery of the BI and specifically the use of motivational interviewing varied between practitioners. A lack of confidence and time constraints were cited as the primary barriers to delivering MI consistent consultations. The LGM resource was reported to be useful and helped guide the consultation and signposting steps.

Risk stratification criteria were used to determine appropriate physical activity options for patients. Only four patients were classified as 'high risk' and were signposted to ER schemes. For 'low' and 'medium' risk patients the most frequently signposted activities were self directed outdoor activities, including active travel and pedometer programmes (n = 131, 44%) and structured leisure centre activities (n = 118, 40%). In discussions however, practitioners expressed concern over the viability of signposting to 'structured activities' due to possible inaccuracies in programmes and timetables.

A total of 101 patients returned for a follow-up consultation, which represents 28% of the 367 patients who were identified as eligible and interested in a brief intervention. It is likely that more patients attended a follow-up appointment however data were missing from one practice (practice #4). Practitioners reported that it was challenging to recall patients for follow-up and this was consistent with their experiences for other interventions designed for preventative purposes as opposed to treatment. In addition, it was viewed as logistically difficult to commence follow-up consultations while still recruiting patients to the intervention.

EMIS data showed that screening and delivery of the BI took on average 20 minutes for patients recruited from the disease registers. These patients received both the screening and BI in the same appointment. Follow-up consultations were estimated to take on average 12 minutes. Practitioners recruiting patients 'opportunistically' reported a wide variation in the time spent screening patients. One GP, for example, spent between 1 and 4 minutes screening patients, while another GP reported between 6 and 23 minutes. Although we observed variation in the time spent screening patients by different health professionals, no clear pattern was observed. The average time spent screening in opportunistic practices was approximately three minutes. Less variation was observed in the BI delivery time and follow-up. The BI took, on average, approximately three minutes and the follow-up consultations took approximately 5 minutes.

## Discussion

Primary care is recognised as a key setting for targeting adults who could benefit from increasing their physical activity levels[3,21]. LGM combines a number of recommended strategies to promote and support behaviour change. The aim of this project was to evaluate the feasibility of implementing LGM in routine primary care practice.

Overall the total number of patients recruited was low, particularly in practices using the opportunistic recruitment method, most likely due to practitioners making their own subjective appraisal of patient suitability, rather than adhering to the screening protocols. However, these findings are consistent with previous research which found that GPs will only opportunistically promote physical activity to patients 'sometimes' or 'occasionally'[22].

Practices recruiting from a disease register achieved higher response rates compared with opportunistic practices, however, the overall recruitment rate of 16% is low in comparison to previous studies which have utilised letters of invitation to recruit patients to physical activity counselling or ER schemes[8,23]. Recruitment rates varied notably between practices. The most successful practice sent additional promotional materials with the letter to patients which may have attracted greater interest in the intervention. In addition, posters and leaflets were displayed at the practice in an attempt to attract more hypertensive patients. It is therefore possible that some hypertensive patients received reinforcement to follow-up on their invitation as a result of the promotional material. These results highlight the benefit of multiple promotional strategies to attract participation in the intervention.

In the remaining two disease register practices, the number and timing of letters affected recruitment rates. These practices sent out a high number of invitations but were unable to respond to the demand for appointments, thus missing the initial interest raised amongst their patients. This highlights the need for careful planning to balance the number of invitations relative to the number of LGM trained staff and the capacity of the practice to respond.

Practitioners supported the use of the GPPAQ as a screening tool, finding it quick and simple to complete and this is consistent with previous research[18]. After completing the GPPAQ, the study protocols required the additional step of reviewing self reported walking and allowed for a revision of the PAI. The probing of walking resulted in 16% of patients being reclassified, all of whom were changed upwards to the 'active' category, thus deeming them ineligible for LGM. Modifications to the GPPAQ scoring is likely to negate the validation of the instrument [24] thus the appropriateness of allowing practitioners to reclassify patients PAI is questionable. An additional concern is that patients identified as 'active' were not directed to 'exit' the pathway as outlined in the protocols, instead they continued through the intervention. This misuses limited resources and suggests that more specific training is needed with clear protocols on how to 'exit' patients classified as 'active'.

Patient interest in LGM was high, with 100% of eligible patients receiving the BI in disease register practices. This was not unexpected as these patients had already expressed interest by responding to their initial invitation. Patient interest amongst those identified as eligible in 'opportunistic' practices was also high, and comparable to other research using a similar recruitment strategy for physical activity counselling in primary care[7]. Practitioner targeting of patients who they perceived to be motivated towards physical activity may also explain this high continuation rate and thus over-estimate the true level of patient response when LGM is delivered on a larger scale.

The time required to deliver preventative services, particularly lifestyle counselling, is a major factor determining implementation. In practices recruiting via the disease registers, screening and BI reportedly took approximately 20 minutes. In contrast, 'opportunistic' practices were notably quicker, taking approximately seven minutes. This suggests that practitioners were able to modify the delivery of the BI to match the available time within a consultation. However, the differences might also reflect variations in both the content and quality of LGM delivery. This is corroborated by the qualitative findings which revealed differences in the delivery of the BI and particularly the use of MI principles and techniques. This raises concern over the consistency with which the intervention was delivered to patients and the extent to which the intervention was true to the goals and theory underpinning its development. Similar issues have been highlighted in previous research [25], as well as the need for physical activity counselling to address issues of treatment fidelity[14]. Although two to three days of training is considered sufficient for understanding the spirit and skills involved in delivering MI, it appears necessary to provide more in-depth and on-going training to support implementation by health practitioners.

The use of supporting resources is well established as part of effective interventions [6] and the LGM materials were positively received and used. Practitioners 'signposted' patients to a variety of opportunities and services within their local community but there was high use of 'self-directed' options due to practitioners' concerns that leisure facility programmes and timetables may not be accurate. The provision of an up-to-date inventory of local opportunities would help support health professionals but such a resource does require on-going resourcing to maintain.

One area that was under-explored in this study was the implementation of LGM alongside or integrated with ER schemes. It is likely that ER schemes were implemented alongside LGM, such that 'high risk' patients were directed to ER schemes using existing procedures rather than first being considered within LGM. This would explain why so few 'high risk' patients were identified through LGM screening. More detailed guidance on how LGM is intended to integrate with, or replace, existing ER protocols is required.

Almost 30% of patients returned for a follow-up appointment. Although this response rate appears low, a systematic review of attendance at ER schemes reported that 80% of participants drop out before the end of the programme which is typically a 3 month period[26]. This finding suggests that this level of attrition may be typical for physical activity interventions in the primary care setting. Although the opportunistic practices appear to be more successful in engaging patients in a follow-up consultation, data were missing from one practice which used the disease register methodology. This reduces our ability to fully understand the actual difference in the response rate at follow-up between the two recruitment methods. Nonetheless, due to the importance of providing follow-up support in helping patients to maintain behaviour change [6], protocols to improve compliance are needed.

This study has several limitations. Firstly, some data were missing from two practices. Consequently, the exact number of patients progressing through all stages of Let's Get Moving could not be determined. This reflects the practical challenges associated with collecting data on the delivery of interventions in a field-based setting. It highlights the need for sufficient training and ongoing support to ensure practitioners adhere to data collection protocols in these types of studies.

In addition, this study focused on assessing feasibility of implementing LGM and did not include the collection of patient level behaviour change data. An effectiveness trial is needed using valid measures of behaviour change over time. Given that the qualitative findings suggest variability in the delivery of the BI and use of MI techniques, further research is warranted to evaluate the actual counselling content and quality of patient-centred discussions at each step of the intervention.

This study was undertaken across six general practices in London, some of which had a high proportion of Asian or Asian British patients, hence these population groups are over-represented when compared to the British population as a whole. Given the lower uptake in these sub populations further formative work and pilot testing may be warranted. In addition, the London-based practices represent a convenient sample and were supportive of the project and motivated to take part. It is unknown what barriers might be associated with delivering LGM in practices which are less motivated, outside of London, or in contexts that have different local opportunities and services for physical activity.

For the purposes of this pilot, recruitment was restricted to two approaches, opportunistic and via the hypertension disease register. The potential to engage patients via other strategies, including new patient registrations, health screening appointments and existing clinics should be explored. Further research is also needed on the effectiveness of utilising other disease registers, as well as the impact of additional promotional materials.

Finally, this study focussed on the delivery of LGM in the primary care setting. Future research could explore alternative delivery models, for example using other allied health professionals such as health trainers to deliver all or part of the intervention.

## Conclusion

This study involved a process evaluation of a systematic approach to physical activity assessment and counselling on physical activity within a primary care setting in the UK. Two approaches to patient recruitment were used ('opportunistic' and disease register) and although notable variations in implementation were observed, practitioners successfully recruited patients to LGM via both methods. Practitioners found it feasible to screen patients on their level of physical activity using the GPPAQ and each component of the brief intervention was delivered to the majority of patients.

Although this process evaluation provides good support for the feasibility of LGM, our results show the need to improve health practitioner adherence to the intervention protocols. Specific issues to address are the use of the GPPAQ to screen and identify all insufficiently active patients and the need for a clear 'exit' protocol for 'active' patients. Also, we recommend more in-depth training and skill development is needed on MI techniques and that practitioners may require on-going support to develop sufficient expertise and confidence to effectively deliver LGM using patient-centred approaches.

In 2009, the English Department of Health launched their 'Be Active Be Healthy' policy which included the phased dissemination of Let's Get Moving[3]. Also, Let's Get Moving has been incorporated within the new National Health Service five-year strategy[27]. Prior to the planned dissemination of LGM, the learning from this pilot should be considered and used to inform the LGM protocols. Furthermore, a robust assessment of effectiveness involving an experimental design and behaviour change measures is warranted.

## Competing interests

The authors declare that they have no competing interests.

## Authors' contributions

FCB developed the study design, provided supervision and guidance throughout the project and assisted in drafting and revising the manuscript. KM developed the data collection tools and supported data collection, undertook the data analysis and assisted in drafting and editing the manuscript. Both authors read and approved the final manuscript.

## Funding

This work was supported by the Department of Health.

## Disclaimer

The views expressed in this publication are those of the authors and not necessarily those of the Department of Health.

## Pre-publication history

The pre-publication history for this paper can be accessed here:

http://www.biomedcentral.com/1471-2458/10/463/prepub
